# Immunization of C57BL/6 Mice with GRA2 Combined with MPL Conferred Partial Immune Protection against *Toxoplasma gondii*

**DOI:** 10.22034/ibj.22.1.22

**Published:** 2018-01

**Authors:** Jalal Babaie, Samira Amiri, Robab Homayoun, Ebrahim Azimi, Reyhaneh Mohabati, Mahboobe Berizi, M. Reza Sadaie, Majid Golkar

**Affiliations:** 1 Molecular Parasitology Lab., Department of Parasitology, Pasteur Institute of Iran, Tehran, Iran; 2 NovoMed Consulting, Silver Spring, Maryland, USA

**Keywords:** Immunization, GRA2, *Toxoplasma*, MPL

## Abstract

**Background:**

We have previously reported that immunization with GRA2 antigen of *Toxoplasma gondii* induces protective immunity in CBA/J (H2k) and BALB/c mice (H2d). We aimed to examine whether immunization of a distinct strain of rodent with recombinant dense granule antigens (GRA2) combined with monophosphorryl lipid A (MPL) adjuvant elicits protective immune response against *T. gondii*.

**Methods:**

C57BL/6 (H2b haplotype) mice were immunized with GRA2, formulated in MPL adjuvant.

**Results:**

Strong humoral response, predominantly of IgG1 subclass and cellular response, IFN-γ, was detected at three weeks post immunization. Mice immunized with GRA2 had significantly (*p* < 0.01) fewer brain cysts than those in the adjuvant group, upon challenge infection. Despite the production of a strong antibody response, IFN-γ production and brain cyst reduction were not significant when the immunized mice were infected four months after the immunization.

**Conclusions:**

We can conclude that GRA2 immunization partially protects against *T. gondii* infection in C57BL/6 mice, though the potency and longevity of this antigen as a standalone vaccine may vary in distinct genetic backgrounds. This observation further emphasizes the utility of GRA2 for incorporation into a multi-antigenic vaccine against *T. gondii*.

## INTRODUCTION

*Toxoplasma gondii* is an obligate intracellular parasite capable of infecting humans, as well as all warm-blooded animals. It is one of the most common human parasites infected about one-third of the world populations[1,2]. Toxoplasmosis is usually benign in immunocompetent individuals; however, primary infection during pregnancy may cause permanent visual and neurological impairments, neonatal malformation, or even death in congenitally-infected newborns. Besides, reactivation of latent infection in the majority of individuals with impaired immune system including AIDS patients results in fatal toxoplasmic encephalitis, which necessitates urgent chemotherapy[1]. Toxoplasmosis is also of considerable economic importance due to the abortion in livestock[2].

Infection with *T. gondii* induces a long-life protective T helper 1 (Th1) immune response, which controls acute infection represented by rapidly dividing tachyzoites and results in encystment of quiescent bradyzoites in tissues[2]. Immune protection is mainly mediated by IFN-γ produced by both CD4^+^ and CD8^+^ T cells, as well as NK cells[3-6]. In addition, CD8^+^ cytotoxic T lymphocytes play a major role in resistance against the infection through cytolysis of infected cells[3,5-7]. An effective vaccine capable of inducing a long-term Th1 response should be able to protect against toxoplasmosis, i.e. the active infection, and to prevent health-threatening complications associated with the infection.

Hundreds of studies were performed during the past five decades to find an effective vaccine against *T. gondii*. The methodologies applied have been improving along with increasing understanding of the host immune response, including the roles of innate and adaptive immune responses in immune protection and scientific innovations such as development of novel adjuvants and DNA recombinant technology[8,9]. The most effective approach for vaccine development has been the use of non-virulent mutated strains of the parasite[8,10]. In fact, a live attenuated vaccine, Toxovax® (Intervet Schering Plough, Boxmeer, The Netherlands), is currently used in sheep to protect against congenital toxoplasmosis, but such a vaccine is not suitable for human use due to the potential risk of reactivation of parasite to the pathogenic form[10,11].

Many studies have exploited vaccine potential of different antigens of *T. gondii* including surface antigens (SAG proteins), bradyzoites-specific antigens, and antigens of the apical organelles, i.e. dense granule antigens (GRA proteins), rhoptries (ROP proteins), and micronemes (MIC proteins)[8]. The GRA antigens are secreted in abundance and constitute major components of both the parasitophorous vacuole surrounding tachyzoites and the cyst wall surrounding the more slowly dividing bradyzoites[12,13]. Several studies have investigated GRA antigens in vaccination surveys and showed that they are capable of inducing protective immune response against *T. gondii*[8,14-16].

Previous studies have indicated that GRA2 is one of the abundant dense granule antigens of *T. gondii* that induces both humoral and cellular responses in humans and can persist for several years[17,18]. Immunization with GRA2 induced a protective immune response against acute[19-24], chronic[25], and congenital[26] infections in mice or rats.

Apart from immunogenic antigen(s), successful vaccination requires a proper adjuvant to enhance both the magnitude and duration of the immune response. Toll-like receptors (TLRs) are innate immune receptors that sense pathogens or vaccines and strongly stimulate the secretion of IL-12 (p70) from dendritic cells that in turn induce Th1 responses. In addition, some TLRs (TLRs 3, 4, 7, and 9) can induce pro-inflammatory cytokines such as IFN-γ and TNF-α that are important for protection against *T. gondii*[24,27]. Monophosphoryl lipid A (MPL), a ligand of TLR4, was the first and the only TLR ligand approved as an adjuvant for human vaccination[28].

We have previously showed that immunization of CBA/J (H2k haplotype) and BALB/c mice (H2d haplotype) with recombinant GRA2 induces a strong Th1 immune response and protects against chronic and acute Toxoplasma infection, respectively[23,25]. A vaccine must be able to induce a stable long-lasting protective immunity in different genetic backgrounds. Lu *et al*.[29] found that C57BL/6 mice are more susceptible to toxoplasmosis than BALB/c and CBA/J mice. In this study, we aimed to examine whether immunization of a distinct strain of rodent (C57BL/6 mice, H2b haplotype) with GRA2 combined with MPL adjuvant elicits protective immune response against *T. gondii*, and if so, whether the elicited immune response provides long-lasting and early stable protection after the immunizations.

## MATERIALS AND METHODS

### 

#### Mice

Female C57BL/6, Swiss, and CBA/J mice, aged 6 to 8 weeks, were obtained from animal center of Pasteur Institute of Iran (Tehran) and maintained under conventional conditions, according to the institutional policies. C57BL/6 mice were used in immunization experiments, and CBA/J mice were used to maintain cysts in their brain. Animal experiments including handling, maintenance, and blood sample collection were approved by Institutional Animal Care and Research Advisory Committee of Pasteur Institute of Iran.

#### Parasites

*T. gondii* tachyzoites of the virulent RH strain were injected into the peritoneal cavity of Swiss mice. Three days after injection, tachyzoites were harvested from peritoneal fluid, washed with PBS and purified through 3.0-µm filters. Cysts of Tehran strain, a type II strain originally isolated from a patient in Tehran, Iran[30], were maintained in CBA/J mice and used to challenge C57BL/6 mice. CBA/J mice were infected intraperitoneally (i.p.) with 100 Tehran cysts (in a volume of 100 to 200 µl). After one month, their brains were removed, homogenized and diluted with PBS. The brain homogenate was injected i.p. into a new group of CBA/J mice.

#### Preparation of toxoplasma lysate antigen (TLA)

RH tachyzoites were sonicated (1 min burst, 1 min cooling, 150 W) in an ultrasonic disintegrator (MSE, Leicester, United Kingdom), centrifuged at 2000 ×g for 30 min, and the protein concentration of the soluble TLA was determined by using the Bio-Rad DC protein assay (Bio-Rad, Hercules, CA, USA). Aliquots of TLA were then stored at -70 °C until use.

#### Production of GRA2

Recombinant GRA2, amino acids 21 to 185, was produced using the gene sequence of RH strain[25]. Briefly, recombinant *Escherichia coli* bacteria were induced with 1 mM isopropyl-D-thiogalactopyranoside (IPTG), centrifuged and resuspended in buffer A (10 mM imidazole, 0.5 M NaCl, 20 mM Tris-HCl at pH 9.0, 0.1% Triton X-100, and proteases inhibitor cocktail without EDTA [Roche, Mannheim, Germany]). The mixture was sonicated at 4 °C and centrifuged at 12,000 ×g at 4 °C for 30 min. The supernatant was recovered, incubated with Ni-nitrilotriacetic acid resin (Qiagen, Courtaboeuf, France) and transferred to an empty column. Following sequential washes of the column with buffers B, C, and D (buffers having the same composition as buffer A but containing 20, 40, and 80 mM imidazole, respectively), the recombinant proteins were eluted with buffer E (buffer having the same composition as buffer A but containing 400 mM imidazole). Purified recombinant GRA2 protein was analyzed by SDS-PAGE, dialyzed against PBS, and stored in aliquots at -20 °C.

#### SDS-PAGE and immunoblotting

SDS-PAGE was performed on 13% polyacrylamide gels[31]. Recombinant GRA2 or tachyzoites was/were boiled in a loading buffer for five minutes, and a portion, equivalent to 5 × 10^10^ tachyzoites or 100 ng of recombinant GRA2, was applied to each lane. The proteins were transferred onto nitrocellulose membranes, saturated with 5% fat-free dried milk in PBS for 1 h and probed with pooled sera of three mice from each animal group diluted at 1:400. Bound antibodies were detected using peroxidase-conjugated rat anti-mouse Ig-kappa light chain antibodies (BD Pharmingen, San Diego, CA, USA). Signals were detected using 3,3-diaminobenzidine tetrahydro-chloride (DAB) tablets (Kem-En-Tec, Copenhagen, Denmark).

#### Study design

**Fig. 1:**
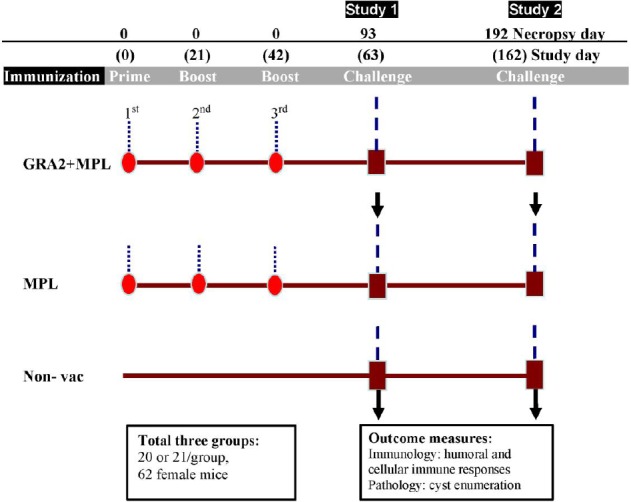
Study design and testing endpoints in C57BL/6 mice. Three groups of mice were included in the study for evaluation of both short-term (study 1) and long-term (study 2) immune protection conferred by GRA2 immunization.

Three groups of C57BL/6 mice, consisted of 20 or 21 mice per group, were included in the study for evaluation of both short-term and long-term immune protection conferred by GRA2 immunization. Mice were injected subcutaneously three times in three-week intervals (Fig. 1). The test group received MPL-formulated GRA2. The control groups consisted of mice receiving MPL or PBS. Three weeks and four months after the last immunization, three mice from each group were euthanized and used for evaluations of the short- and long-term humoral and cellular immune responses, respectively. At the same time, seven or eight mice per group were infected with tissue cysts, and brain cysts were enumerated one month later.

#### Immunization of C57BL/6 mice

Female C57BL/6 mice, 6 to 8 weeks old, were injected subcutaneously in their hind footpad three times, at weeks 0, 3, and 6 with 20 µg of GRA2 formulated in Sigma adjuvant System (Sigma, St. Louis, MO, USA) in a volume of 50 µl. Briefly, each vial of adjuvant, containing 0.5 mg of both MPL from Salmonella minnesota and synthetic trehalose dicorynomycolate in 2% oil (squalene), Tween 80, and water was reconstituted with 1 ml of GRA2-saline solution (400 µg/ml) according to the manufacturer’s instructions and administered to mice as 25 μg MPL per dose[32]. The blood samples from three mice in each group were collected by retro-orbital puncture three weeks and four months after the last immunization, and sera were stored at -20 °C until use.

#### Enzyme linked immunosorbent assay (ELISA)

Maxisorp multiwell plates (Nunc, Roskilde, Denmark) were coated overnight at 4 °C with 10 µg/ml of TLA in 50 mM carbonate buffer (pH 9.6). The plates were washed with PBS containing 0.05% Tween 20 (PBS-T), and blocked with 200 µl blocking buffer (PBS-T containing 1% BSA) at 37 °C for 1 h. Subsequently, the plates were incubated with 100 µl of sera, taken from immunized mice at three weeks or four months post immunization, diluted at 1:100 in a blocking buffer at 37 °C for 1 hour. The plates were washed and incubated with 100 µl HRP-conjugated rabbit anti-mouse IgG, IgG1, and IgG2a/c (1:10,000, 1:3,000, and 1:3,000, respectively; Zymed, South San Francisco, CA, USA) at 37 °C for 1 h. Finally, the enzymatic activity was revealed using tetramethylene benzidine dihydrochloride (Kem-En-Tec, Copenhagen, Denmark). After 12 min of incubation at room temperature, the reaction was stopped by adding 100 µl of 2 M H_2_SO_4_. The optical density (OD) value was measured at 450 nm by an ELISA reader (BioTek Instruments, Highland Park, VT, USA), with a reference wavelength of 630 nm. All samples were run in duplicates.

#### Cytokine assays

The spleens from three mice per group were removed aseptically three weeks or four months after the last immunization, and spleen cell suspensions were prepared by squeezing the whole organs in Red Blood Cell Lysing Buffer (Sigma, St. Louis, MO, USA). Spleen cells were resuspended in DMEM medium supplemented with 10% fetal calf serum. The cells (3 × 10^10^ cells/well) were seeded in triplicates in flat-bottom 96-well microtiter plates and cultured with TLA (15 µg/ml) and concavalin A (5 µg/ml; both from Sigma, St. Louis, MO, USA), as positive controls or medium alone (negative control) with 5% CO_2_ at 37 °C for 3 days. Cell-free supernatants were harvested for IL-2 and IL-10 at 24 h and for IFN-γ at 72 h[25]. Concentrations of the three cytokines in culture supernatants were determined using ELISA kits (Mabtech, Stockholm, Sweden) as described by the manufacturer. The lower limits of detection were 5, 15, and 20 pg/ml for IFN-γ, IL-2, and IL-10, respectively. All assays were performed in duplicates, and the results were expressed as means ± SD for each group.

#### Challenge infection

C57BL/6 mice were infected i.p. with 20 brain cysts of Tehran strain either three weeks or four months after the last immunization. One month later, the mice were sacrificed, and their brains were removed. Cysts were obtained by homogenizing each brain in 2 ml PBS. The mean number of cysts per brain was determined microscopically by counting four samples (20 µl each) of each homogenate. The results were expressed as mean ± SD for each group.

### Statistical analysis

The mean of each variable (total IgG, IgG1, IgG2a/c, IFN-γ, IL-2, IL-10, and cyst numbers) was compared between the different groups using one-way ANOVA, followed by Tukey’s HSD, as the post-hoc test. All statistical analyses were performed using the SPSS software version 18 (SPSS, Inc.). The SD represents the mean value of individually evaluated mice. Two-sided *p* values <0.05 were considered to indicate statistical significance.

## RESULTS

### 

#### Immunization with GRA2 induces strong IgG antibody response which lasts for four months

**Fig. 2:**
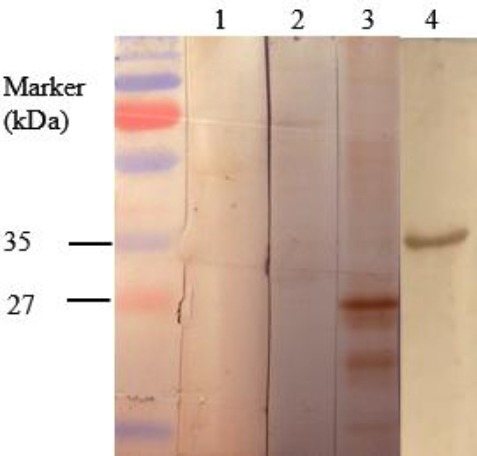
Immunoblotting of GRA2-immunized mice. Immuno-blot of RH tachyzoite antigens probed with pooled sera from three mice injected with PBS (lane 1), injected with MPL (lane 2) or immunized with GRA2 + MPL (lane 3). Pooled sera from three mice immunized with GRA2 + MPL was also used to probe recombinant GRA2 (lane 4).

To determine whether the immunized mice developed specific anti-*T. gondii* IgG antibody response, blood samples of three mice in each group were obtained three weeks and four months after the last immunization, and the sera were tested using immunoblotting and ELISA. On immunoblots, pooled sera, obtained from GRA2-immunized mice at short-term and long-term immunization (data not shown), reacted with both recombinant GRA2 and native GRA2 present in tachyzoites. Recombinant GRA2 was seen at about 35 kDa due to the presence of 44 extra aminoacids, compared to native GRA2, including two histidine tags[25] (Fig. 2). The smaller protein bands observed in the blot are probably degradants of GRA2. Sera from the adjuvant group or PBS-injected mice did not recognize any specific protein band in tachyzoites or GRA2-loaded lanes.

**Fig. 3:**
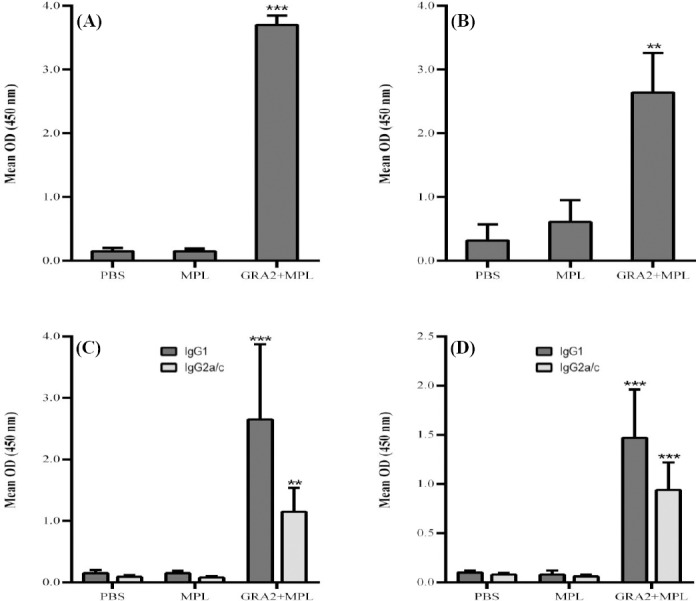
Determination of specific anti-*T. gondii* IgG antibodies in the sera of immunized mice. Determination of specific total and subclass anti-*T. gondii* IgG antibodies in the sera of C56BL/6 mice immunized with GRA2 + MPL, injected with MPL or PBS at short-term (A and C) or long-term (B and D) immunization. Results were expressed as the mean ± SD. ^**^ *p* < 0.01 and ^***^ *p* < 0.001, compared to MPL group (ANOVA test).

Immunization with GRA2 predominantly elicits the production of long-lasting IgG1 and IgG2a/c antibodies. In ELISA experiments, GRA2-immunized mice exhibited strong IgG response at three weeks post immunization, which declined a bit at four months post immunization (Fig. 3A and 3B).

As reported previously, C57BL/6 mice with the Igh-1b allele do not have the gene for IgG2a, but rather the IgG2c isotype[33-35]. However, many studies including the present study, detected IgG2a in C57BL/6 mice[36-41]. This result is probably due to the cross-reaction of IgG2a detecting antibodies with IgG2c[38]. In our study, we referred to these isotypes as IgG2a/c.

The production of specific IgG1 and IgG2a/c subclass antibodies were measured by ELISA at three weeks and four months post immunization. C57BL/6 mice produced preferentially IgG1 antibodies in response to immunization with GRA2 at both time points, although IgG2a/c antibodies were also produced at substantial amount (IgG2a/c: IgG1 = 0.4; Fig. 3C and 3D). These results thus suggested that the specific immune response induced in GRA2-immunized mice is a mixed Th1/Th2 response.

#### Mice immunized with GRA2 produce IFN-γ only at three weeks post immunization

**Fig. 4:**
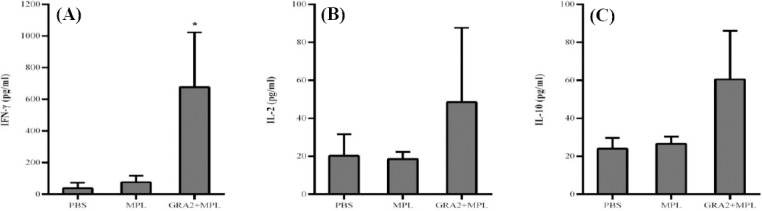
Analysis of cytokine production in spleen cell culture of mice after restimulation with TLA. Spleen cells were recovered from three mice per group. Cells (3 × 10^10^) were seeded per well and stimulated with TLA at the concentration of 15 µg/ml. Tthe levels of IFN-γ (A), IL-2 (B), and IL-10 (C) were measured in cell supernatants 24 h (IL-2 and IL-10) or 72 h (IFN-γ) after restimulation. Results were expressed as the mean ± SD of duplicate wells. * indicates a difference with the MPL group (*p* < 0.05).

To further characterize the nature of the immune response elicited by GRA2 immunization, spleens of three mice in each group were removed three weeks and four months post immunization, and spleen cell preparations were stimulated with TLA. Cell culture supernatants were harvested at 24 h for detection of IL-2 and IL-10, and at 72 h for the measurement of IFN-γ. The results showed that at three weeks post immunization the spleen cells of GRA2-immunized mice produced IFN-γ more than 11 times as high as those produced by spleen cells of the MPL group (*p* < 0.05; Fig. 4A). In addition, spleen cells of GRA2-immunized mice produced about three times more IL-2 and 2.5 times more IL-10, compared to the MPL group; however, their productions were not significant (*p* = 0.1 for IL-2 and 0.12 for IL-10; Fig. 4B and 4C). In contrast, no significant amounts of IFN-γ, IL-2, and IL-10 were produced by mice at long-term immunization (data not shown).

#### Immunization with GRA2 decreases brain cyst production only at three weeks post immunization

**Fig. 5:**
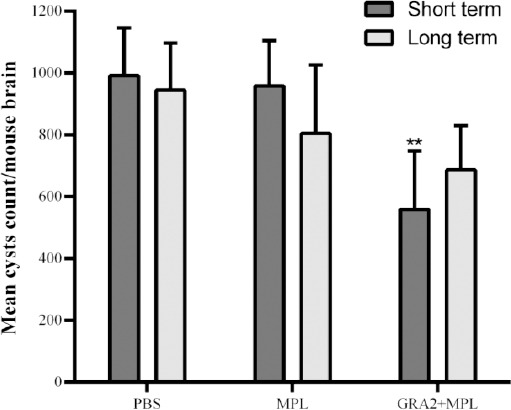
Short-term and long-term protective effects of immunization with GRA2 combined with MPL in C57BL/6 mice. Mice immunized with GRA2 + MPL, MPL or PBS were infected intraperitoneally three weeks or four months after the last immunization with 20 Tehran cysts. Their brain cyst loads were determined one month later and compared to those of control groups. The results were expressed as the total number of brain cysts ± SD calculated from four counts of 20-µl samples from the total brain homogenate. The graph represents that the results obtained from one of the two experiments that provided similar results. ** indicates a difference with the MPL group (*p <* 0.01).

Three weeks after the immunization, seven or eight mice were challenged with 20 freshly prepared brain cysts of *T. gondii*. One month later, the animals were euthanized, and the brain cyst burden was measured by microscopy. The results showed that the mice immunized with GRA2 decreased brain cysts production by 37.1%, compared to the MPL group (*p* < 0.01; by 44% as compared to the PBS group; Fig. 5). To evaluate the long-term protection, seven or eight mice from each group were infected four months after the last booster and before infectious challenge, and the brain cysts were counted one month later. The results showed that fewer brain cysts were developed in the GRA2-immunized mice than either the PBS or the MPL group, though the reduction was not significant (*p* = 0.32; Fig. 5).

## DISCUSSION

In this study, we report that immunization with GRA2 in combination with MPL induces strong humoral and cellular immune responses of a mixed Th1/Th2 type, and confers short-term protective immunity in C57BL/6 mice. Though a strong humoral response was detectable at four months post immunization, the cellular response and immune protection diminished over time falled below the significant level at four months post immunization.

The IgG response in ELISA was not greatly decreased at long-term evaluation. Similar OD values of IgG antibodies observed at short- and long- term immune evaluation suggest strong induction of B cells and long-lasting production of IgG antibodies, otherwise one should expect great decrease (as much as 30 times) of IgG1 and IgG2a titers at four months post immunization regarding approximately three weeks half-life of them.

We observed that IgG1 subclass antibodies were produced more than IgG2a/c antibodies, suggesting a mixed Th1/Th2 response as IgG2a/c and IgG1 are markers of Th1 and Th2 immune responses, respectively[24]. Similarly, immunization of BALB/c with GRA2 in Freunds’ adjuvant induced more IgG1 than IgG2a[24]. However, the ratio of IgG2a/IgG1 was slightly higher in BALB/c mice immunized with GRA2 adsorbed on Poly (DL-lactide-co-glycolide) microspheres[23]. In addition, we previously showed that immunization of CBA/J (H2k haplotype) with GRA2 combined with MPL resulted in a high ratio of IgG2a/IgG1 antibodies[25]. Discrepancy between these results might be due to the different adjuvant/delivery systems, as well as mice strain used in these studies[42]. To examine more precisely the immune response elicited by immunization with GRA2, the levels of IFN-γ, IL-2, and IL-10 were measured in the spleen

cell cultures at short- and long-term time points. GRA2-immunized mice produced elevated levels of IFN-γ at short-term evaluation, indicating a Th1 response. This is intriguing because IFN-γ is the main mediator of protection against Toxoplasma infection. Similarly, immunization of CBA/J mice with GRA2 induced high levels of IFN-γ[25]. The production of IL-2 and IL-10 was hardly significant, though indicating a trend toward antigen-specific increase. The production of IL-10 is important in the regulation of host immune response against *T. gondii* as it prevents fatal inflammatory pathology induced by exacerbated IFN-γ production in C57BL/6 mice[43,44]. In contrast, we were not able to detect any specific IFN-γ, IL-2, and IL-10 at long-term immune evaluation. This means that immunization with GRA2 formulated in MPL was not able to induce sufficient durable memory T cells to secrete detectable amounts of the cytokines.

Consistent with the increased production of IFN-γ, GRA2 immunization decreased brain cysts development by 37.1%, compared to MPL group when the challenge infection was performed three weeks after the last immunization. We formerly reported that immunization of CBA/J mice with GRA2 formulated in MPL decreased the brain cysts production by 69.8%[25]. Moreover, in our previous study, we demonstrated that GRA2 adsorbed on Poly (DL-lactide-co-glycolide) microspheres induced strong humoral and cellular response in BALB/c mice and protected against acute infection[23]. The possible explanations for the lower protective efficacy observed in the present study could be the lower immune-genicity of GRA2 in C57BL/6 mice, inherent incapability of C57BL/6 mice to fully control the infection[45,46], and the difference in virulence of the *T. gondii* strain[30] used for the challenge infection. In fact, the efficacy of different vaccination strategies using sub-unit vaccines for prevention of cyst production varies greatly, and several studies have achieved protective efficacies similar to the present study[8,47-49]. Bastos *et al*.[50] selected two overlapping short peptides of GRA2, based on their reactivity with a monoclonal antibody against GRA2, fused them to bovine serum albumin and evaluated their immunogenicity and protective efficacy in C57BL/6 mice using the alum adjuvant. No reduction in brain cysts production was observed when parasite burden was measured by real-time PCR and by direct cyst count using optical microscopy. However, combination of the two peptides was reportedly able to increase the survival rate of mice during acute infection. We used the whole GRA2 sequence, without the signal sequence, formulated in MPL for immunization of C57BL/6 mice and showed significant protection against brain cysts production. The different protection against brain cyst production observed in our study and those of Bastos et al., might be the use of whole GRA2 protein and/or potent Th1-directing MPL adjuvant in our study.

In the current study, we decided to examine long-term immune protection induced by GRA2 + MPL since previous studies have reported the induction of long-lasting immunity by GRA2 in humans[17] and mice[22]. Vaccination of Swiss-Webster mice with F3G3 antigen, which is composed of GRA2 (P28) and a 58-kDa component[51], in Freund’s adjuvant conferred long-term protection against lethal challenge with *T. gondii* tachyzoites[22]. Furthermore, Prigione *et al*.[17] investigated the role of GRA2 in the maintenance of long-term T cell response against *Toxoplasma*, and found that 6 out of 25 CD4^+^ tachyzoite-specific T cell clones proliferated to purified GRA2. MPL has also been indicated to be capable of promoting durable humoral and cellular immune response[52,53]. Despite the immune protection observed at three weeks post immunization, reduction in brain cysts was not significant when the immunized mice were infected four months after the immunization. Based on our knowledge, long-term protective immunity depends on the development of long-term memory T cells from effector T cells, which is influenced by several factors including antigen exposure, co-stimulation, and the level of inflammation[54]. The lack of long-term immune protection in our study might be explained by poor differentiation of effector T-cells to long-term memory T-cells. Nevertheless, the lack of long-term protection in the present study does not exclude the possible capability of GRA2 in inducing long-lasting immune response in other strains of mice or in humans, as previously described[17,22]. On the other hand, it might be possible to prolong the immune protection through applying different vaccination strategies, different adjuvants, and novel vaccine delivery systems[55-57].

MPL, a ligand of toll-like receptor 4, is known to stimulate IFN-γ and influence development of Th1 response, which is essential for protection against *T. gondii*. However, a number of studies have shown that MPL potentiates both Th1 and Th2 response and stimulates a mixed Th1/Th2 response[58,59]. The presence of TLR4 ligands, such as glycosyl-phosphatidylinositols, and heat shock protein 70[60-62] propose a role for TLR4 in the induction of protective immune response to *T. gondii*.

Several investigations have reported the efficacy of various forms of GRA2 as native protein[19,63], recombinant protein[20,25], DNA vaccine[21], and multi-antigenic DNA vaccine[64] in eliciting protective immunity against acute, chronic and congenital Toxoplasma infection. Native purified GRA2 conferred protection against both acute and congenital toxoplasmosis[19,22,25,63]. Others showed that a GRA2-SAG1 chimeric protein[20] and multi-antigenic DNA vaccines containing GRA2 epitopes[64,65] prolonged the survival of immunized mice; however, the relative contribution of each antigenic portion in the protective immunity was not investigated. Recently, Zhou *et al*.[21] have demonstrated that DNA vaccine comprising GRA2 is capable of increasing survival time of immunized mice.

The fact that GRA2 is able to induce protective immune response in both CBA/J and C57BL/6 mice is encouraging since a vaccine antigen must be able to provide protection in different genetic backgrounds. The combination of GRA2 with other immunogenic antigens of *T. gondii* such as SAG1 might be able to induce a better protection against the infection[21]. Although a desirable feature of a vaccine is required to confer a long-lasting protective immunity, this component of *T. gondii* antigens may provide useful early protection. It might be possible to enhance the efficacy by using improved adjuvant(s) and/or methods of vaccine delivery that mimic the native infection.
